# The effect of *Torreya grandis* inter-cropping with *Polygonatum sibiricum* on soil microbial community

**DOI:** 10.3389/fmicb.2024.1487619

**Published:** 2024-12-04

**Authors:** Quanchao Wang, Xiaojie Peng, Yuxuan Yuan, Xudong Zhou, Jianqin Huang, Haonan Wang

**Affiliations:** State Key Laboratory of Subtropical Silviculture, College of Forestry and Biotechnology, Zhejiang A & F University, Hangzhou, Zhejiang, China

**Keywords:** plant health, soil microbiota, agroforestry, sustainable development, soil microbial function

## Abstract

**Background:**

Inter-cropping is a reasonable planting pattern between different plants. Inter-cropping of *Torreya grandis* with *Polygonatum sibiricum* is a relatively mature planting pattern in China, which has been applied to improve soil ecological environment and reduce the occurrence of pests and diseases in China. However, there is currently limited knowledge on the response of soil microbial communities to this practice.

**Methods:**

In this study, we employed Illumina MiSeq sequencing coupled with Functional Annotation of Prokaryotic Taxa (FAPROTAX) and Fungi Functional Guild (FUNGuild) analyses to investigate the dynamic changes in soil microbial communities across seven treated groups [the bulk soil of the *T. grandis* inter-cropping with *P. sibiricum* (IB), the bulk soil for mono-cropping of *P. sibiricum* (PB), the bulk soil for mono-cropping of *T. grandis* (TB), the *P. grandis* rhizosphere soil of the *T. grandis* inter-cropping with *P. sibiricum* (IPR), the rhizosphere soil for mono-cropping of *P. sibiricum* (PR), the *T. grandis* rhizosphere soil of the *T. grandis* inter-cropping with *P. sibiricum* (ITR), and the rhizosphere soil for mono-cropping of *T. grandis* (TR)].

**Results:**

The results showed that the rhizosphere soil of *Torreya-Polygonatum* inter-cropping exhibited higher microbial community richness, diversity and evenness than mono-cropping (ITR > TR, IPR > PR). Inter-cropping increased the abundance of Micrococcaceae, Xanthobacteraceae, *Saitozyma*, while decreased *Bacillus, Burkholderia, Streptomyces, Cladosporium*, and *Gibberella* significantly of the rhizosphere soil of *T. grandis*. Further, the abundance of pathogens, such as *Fusarium* and *Neocosmospora*, was higher in mono-cropping samples compared to inter-cropping. There existed distinct variations in bacterial and fungal communities among all groups except for IB and TB. The FAPROTAX and FUNGuild analyses results indicated that inter-cropping significantly enhanced soil microbial function associated with nutrient cycling and exhibited a consistent increase in the relative abundance of nitrogen-cycling and carbon-cycling bacteria, and decreased the abundance of plant pathogen guild in the inter-cropping sample ITR compared to the mono-cropping TR.

**Conclusion:**

Our findings suggest that *T. grandis* inter-cropping with *P. sibiricum* not only enhance the diversity of soil microbial communities, but also improve the nitrogen and carbon cycling functions. In addition, the inter-cropping can effectively reduce the relative abundance of some soil-borne pathogens for *T. grandis* and *P. sibiricum*, indicating that this intercropping method may alleviate the impact of pathogens on crops, thus providing assistance for plant disease prevention and sustainable management.

## 1 Introduction

Plant rhizosphere communicating with soil microorganisms represents the important site for the interaction between microbes and plant hosts (Hinsinger et al., 2009). The host can reshape the microbial community structure through root exudates and soil composition has effect on rhizosphere microbial communities (Haichar et al., 2008; Berg and Smalla, 2009). Higher microbial diversity enhances nutrient exchange and resource utilization, and increases plant productivity (Tedersoo et al., 2020). The reduction of soil biodiversity, however, significantly impairs the functioning of ecosystems (Wagg et al., 2014).

Plant cropping practice impacts plant growth, soil physicochemical properties and microbial community. Long-term cropping of the same plant species leads to the decrease of soil enzyme activity, deterioration of physiochemical properties, and change of the soil microbial structure and diversity (Machado et al., 2007; Du et al., 2017; Yu et al., 2017; Bai et al., 2019; Pervaiz et al., 2020). It thus reduces plant growth and yield, and promotes damage from pests, diseases and weeds (Zhang et al., 2017; Zeng et al., 2020). In contrast, inter-cropping of different plant species can improve soil micro-ecological environment by recruiting beneficial microbiota (Li et al., 2007; Wang et al., 2021). The mustard inter-cropping with cucumber increased the diversity of soil microbiota and the abundance of beneficial microorganisms (Li and Wu, 2018). The peanut-tobacco inter-cropping improved soil ecology and microbial structure, increasing the number of beneficial bacteria and decreasing the number of pathogenic bacteria (Gao et al., 2019). Plant inter-cropping can also inhibit the spread of soil-borne diseases such as *Fusarium* wilt and *Phytophthora* blight by changing the content and composition of root exudates and increasing the diversity of rhizosphere microbial community structure (Yu et al., 2019; Lv et al., 2020; Zhang et al., 2020; Song et al., 2022).

*Torreya grandis cv Merrillii* (Taxaceae, *Torreya*) is a precious economic tree cropped in subtropical mountains in China with various uses such as for fruit, medicine, oil, wood and ornamental purposes. The root system of *T. grandis* exhibits characteristics such as shallow depth, wide distribution, and strong adaptability to drought and nutrient-poor soil conditions. Furthermore, the afforestation process minimizes any potential damage to existing vegetation, rendering it highly suitable for cultivation in forested areas (Chen and Jin, 2019; Chen and Chen, 2021). The perennial herbaceous plant, *Polygonatum sibiricum* (Asparagaceae *Polygonatum*) is a perennial tuberous herb with shallow roots, which can be taken as food, and is rich in chemical components beneficial to human health (Kato et al., 1994; Xian et al., 2012; Luo et al., 2018). Inter-cropping of *T. grandis* with *P. sibiricum* has thus been applied in China for efficient land utilization and yield increase, thereby facilitates the rapid establishment of young *Torreya* forests, thereby expediting forest formation while also yielding short-term economic benefits, and effectively mitigating soil and water erosion resulting from excessive planting (Luo et al., 2023). However, its effect on soil microbial community remains unexplored.

In this study, we collected the rhizosphere and bulk soil samples of inter-cropping and mono-cropping of *T. grandis* and *P. sibiricum*. The high-throughput Illumina MiSeq sequencing coupled with Functional Annotation of Prokaryotic Taxa (FAPROTAX) and Fungi Functional Guild (FUNGuild) analyses were used to address: (a) The effects of *Torreya-Polygonatum* inter-cropping on the composition of rhizosphere and bulk soil bacterial and fungal communities comparing to mono-cropping; (b) The response and function of *Torreya-Polygonatum* inter-cropping to bacterial and fungal communities.

## 2 Materials and methods

### 2.1 Site description, experimental design, and soil sample collection

The study areas were located in Chun'an County, Hangzhou, China (29°11′46″N, 118°42′10″E), from where there is subtropical monsoon climate, characterized by high temperature and rainy summer, and mild winter with little rain. The meteorological parameters there were obtained from the China Meteorological Data Service website (http://data.cma.cn/). The average annual sunshine and precipitation are 1,850.3 h and 1,515.1 mm with average temperature of 17.2°C ranging from −5.4°C in winter to 37.6°C in summer. The *T. grandis* plantation was established in 2011, and it has been inter-cropped with *P. sibiricum* since 2015 (Supplementary Table 1).

**Figure 1:**
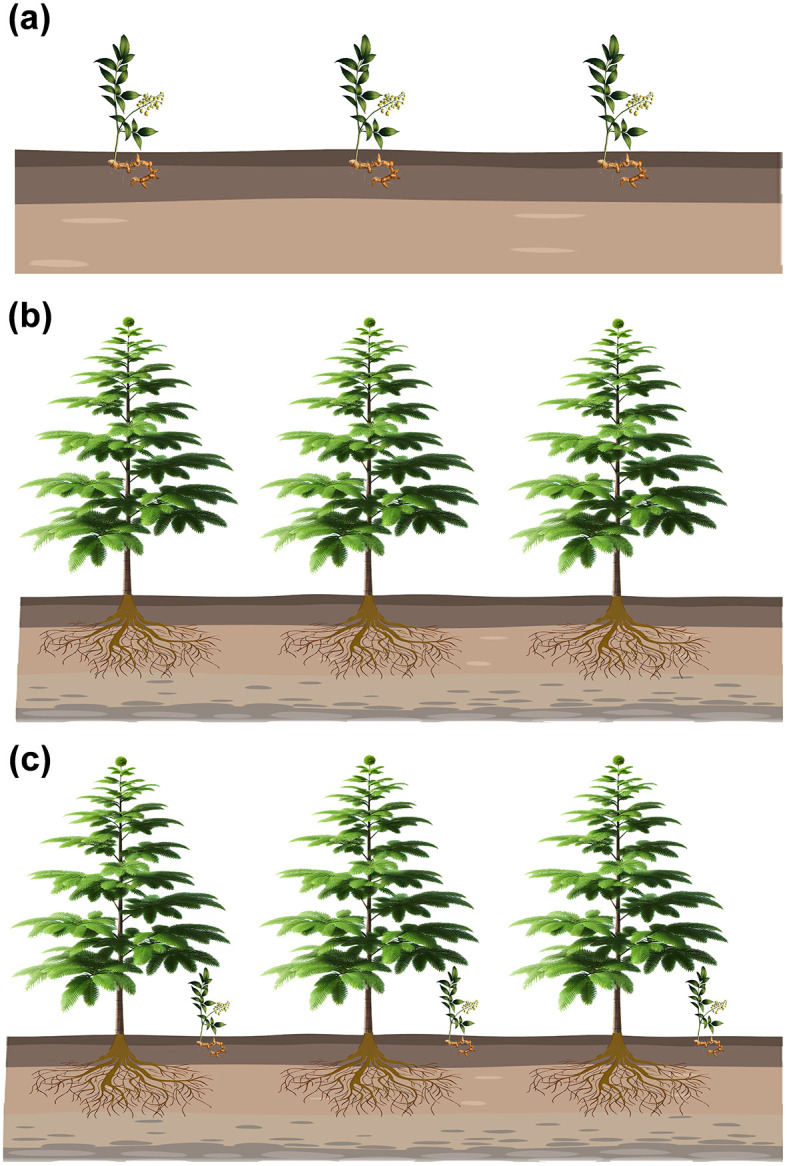
Schematic diagram showing different cropping practice. **(a)** *Polygonatum sibiricum* mono-cropping. **(b)** *Torreya grandis* mono-cropping. **(c)** Inter-cropping of *P. sibiricum* and *T. grandis*.

Seven different sets of samples including the rhizosphere and bulk soil were collected from inter-cropping and mono-cropping of *T. grandis* and *P. sibiricum* (Figure 1): the bulk soil of the *T. grandis* inter-cropping with *P. sibiricum* (IB), the bulk soil for mono-cropping of *P. sibiricum* (PB), the bulk soil for mono-cropping of *T. grandis* (TB), the *P. grandis* rhizosphere soil of the *T. grandis* inter-cropping with *P. sibiricum* (IPR), the rhizosphere soil for mono-cropping of *P. sibiricum* (PR), the *T. grandis* rhizosphere soil of the *T. grandis* inter-cropping with *P. sibiricum* (ITR), and the rhizosphere soil for mono-cropping of *T. grandis* (TR).

The soil samples were collected during the maturation season of *T. grandis* and *P. sibiricum* (September 22, 2022), and arranged in a randomized block design (3 replications). The bulk soil samples were collected using four-point sampling method to select four square points at a distance of 10 cm away from the plant roots, and passed through a 2-mm sieve to remove plant roots and debris. The rhizosphere soil samples were collected from the roots of *T. grandis* and *P. sibiricum* and rinsed with phosphate buffer solution (PBS; McPherson et al., 2018).

### 2.2 Soil DNA extraction, PCR amplification, and high-throughput sequencing

Soil DNA was extracted using the Soil DNA kit (OMEGA BIO TEK) following the manufacturer's instruction. DNA concentration and quality were detected by 1% agarose gel electrophoresis with NanoDrop 2000 spectrophotometer (Thermo Fisher Scientific Inc., USA).

The bacterial V3-V4 variable region was amplified with primers 338F (5′-ACTCCTACGGGAGGCAGCAG-3′) and 806R (5′-GGACTACHVGGGTWTCTAAT-3′; Xu et al., 2016). The fungal ITS region was amplified with primers ITS3F (5′-GCATCGATGAAGAACGCAGCGCATCGATGAAGAACGCAGC-3′) and ITS4R (5′-TCCTCCGCTTATTGATATGC-3′; Toju et al., 2012). The PCR reaction condition was as follows: initial denaturation at 95°C for 3 min, 27 cycles of denaturation at 95°C for 30 s, annealing at 55°C for 30 s, 72°C for 30 s, and then a final extension of 10 min at 72°C. The PCR amplification products were mixed and detected by 2% agarose gel electrophoresis. Amplicons were extracted using QuantiFluor™-ST (Promega) and quantified using the AxyPrep DNA Gel Extraction Kit (Axygen Biosciences, Union City, CA, U.S.) according to the manufacturer's protocol. The PCR product was sequenced with paired-end (PE = 300) Illumina MiSeq platform at Majorbio (Shanghai International Medical Zone). The raw reads were deposited in the Sequence Read Archive database maintained by the National Center for Biotechnology Information (NCBI, Accession Number: PRJNA1150097).

The raw sequences were denoised, quality controlled and clustered into Amplicon Sequence Variant (ASV). The optimized data were then processed using Sequence Denoising Methods (DADA2/Deblur, etc.) to obtain ASV representing sequence and abundance information. Based on the representative ASV sequence and abundance information, analyses including microbial taxa, community diversity, species difference, correlation, and functional prediction were performed.

### 2.3 Statistical analysis

The bacterial and fungal community richness (Chao1), α-diversity index (Shannon), evenness (Shannoneven), and Pd (Phylogenetic diversity) were calculated using QIIME (Version 1.7.0). The bacterial and fungal indicator taxa were identified using the linear discriminant analysis Effect Size (LEfSe). LEfSe uses non-parametric Kruskal-Wallis rank sum tests to detect significant features and performs LDA to estimate the effect size of each feature (*P* < 0.05, and LDA score > 3.5). Principal coordinate analysis (PCoA) based on Bray–Curtis similarities at ASV level were used to calculate the difference in soil microbial community composition (beta diversity) by R (R v.3.4.4) program. Microbial networks diagrams were drawn using the Gephi software. Ecological function prediction of microorganisms b was performed using the FAPROTAX database (Louca et al., 2016) for bacteria (16s gene) and the FUNGuild database (Nguyen et al., 2016) for fungi (ITS gene).

## 3 Results

### 3.1 Bacterial and fungal community diversity in bulk and rhizosphere soil under different cropping patterns

**Figure 2:**
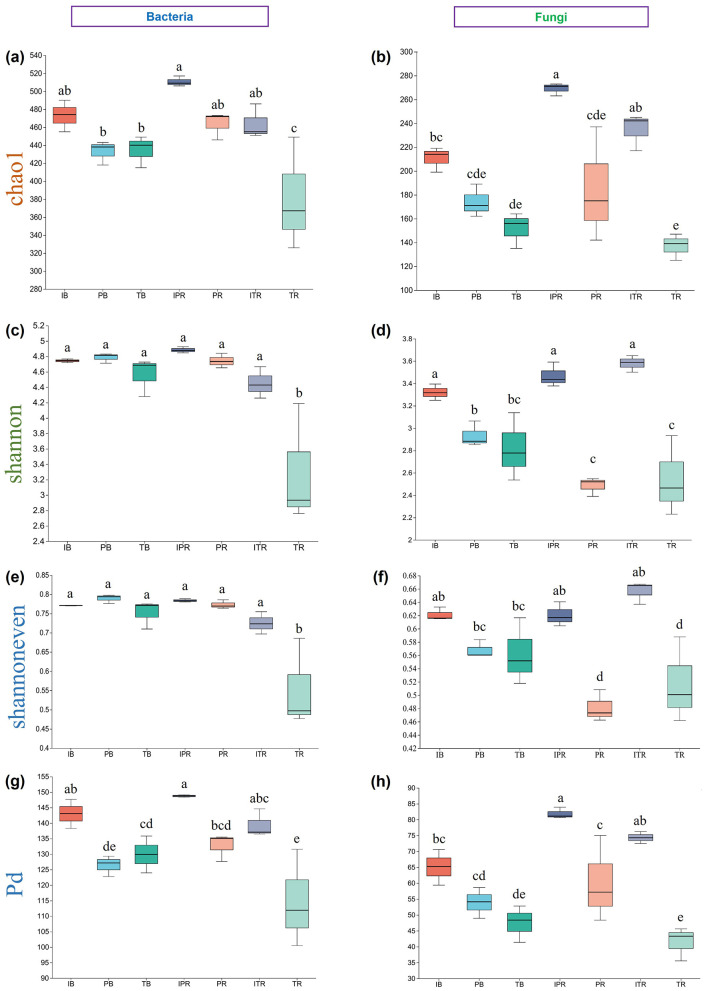
Alpha diversity of bacterial and fungal in bulk soil. **(a, b)** Chao1 index in **(a)** bacteria and **(b)** fungi. **(c, d)** Shannon index in **(c)** bacteria and **(d)** fungi. **(e, f)** Shannoneven diversity index in **(e)** bacteria and **(f)** fungi. **(g, h)** Pd diversity index in **(g)** bacteria and **(h)** fungi. Different lowercase letters indicate significant differences among the seven sets of samples. IB: the bulk soil of the *T. grandis* inter-cropping with *P. sibiricum*, PB: the bulk soil for mono-cropping of *P. sibiricum*, TB: the bulk soil for mono-cropping of *T. grandis*, IPR: the *P. grandis* rhizosphere soil of the *T. grandis* inter-cropping with *P. sibiricum*, PR: the rhizosphere soil for mono-cropping of *P. sibiricum*, ITR: the *T. grandis* rhizosphere soil of the *T. grandis* inter-cropping with *P. sibiricum*, and TR: the rhizosphere soil for mono-cropping of *T. grandis*.

Results showed that there were significant differences among the samples (*P* < 0.05) for bacterial and fungal community richness (Chao1), community diversity (Shannon), community evenness (Shannoneven), and phylogenetic diversity (PD). The rhizosphere soil of *T. grandis* mono-cropping (TR) exhibited the lowest bacterial and fungal community richness (381.70 ± 36.70; 136.47 ± 6.45) as well as phylogenetic diversity (114.25 ± 9.07; 41.48 ± 3.05), along with a lower bacterial community diversity (3.30 ± 0.45) and bacterial community evenness (0.55 ± 0.066; Figure 2, Supplementary Table 2). In contrast, the rhizosphere soil of *Torreya-Polygonatum* inter-cropping samples had higher fungal Chao1, Shannon, Shannoneven, and PD indices than those of mono-cropping (IPR > PR, ITR > TR). These results were consistent with the findings observed in the ITR and TR samples for bacterial communities. However, inter-cropping had minimal impact on the composition of rhizosphere soil bacterial community. Further, the bulk soil of the *T. grandis* inter-cropping with *P. sibiricum* (IB) had the higher fungal community richness, diversity, evenness and Phylogenetic diversity than the bulk soil of *T. grandis* (TB) and *P. sibiricum* (PB), respectively. There was no significant difference in bacterial richness, diversity, evenness and phylogenetic diversity between the rhizosphere and bulk soil samples in the *T. grandis* inter-cropping with *P. sibiricum* and *P. sibiricum* (IB and IPR, ITR; PB and PR), while the rhizosphere soil for mono-cropping of *T. grandis* were lower than bulk soil (TB and TR). After inter-cropping, the richness and phylogenetic diversity of the inter-cropping rhizosphere soil of *P. sibiricum* were significantly higher than bulk soil (IB and IPR), while no significant difference for *T. grandis* (IB and ITR). Moreover, there was no significant difference in fungal richness and phylogenetic diversity between the bulk soil and the rhizosphere soil for mono-cropping of *T. grandis* and *P. sibiricum* (PB and PR; TB and TR; Figure 2, Supplementary Table 2).

### 3.2 Bacterial and fungal community composition at ASV level under different cropping patterns

All obtained sequences were classified to bacterial and fungal domains. Among them, 74.7% were classified to 39 bacterial phyla and 1,003 genera, and 79.8% were classified to 13 fungal phyla and 545 genera. The bacterial and fungal community coverage is 99.96–100% (Supplementary Table 2), indicating that the sequence data reasonably reflect the species and basic structure.

**Figure 3:**
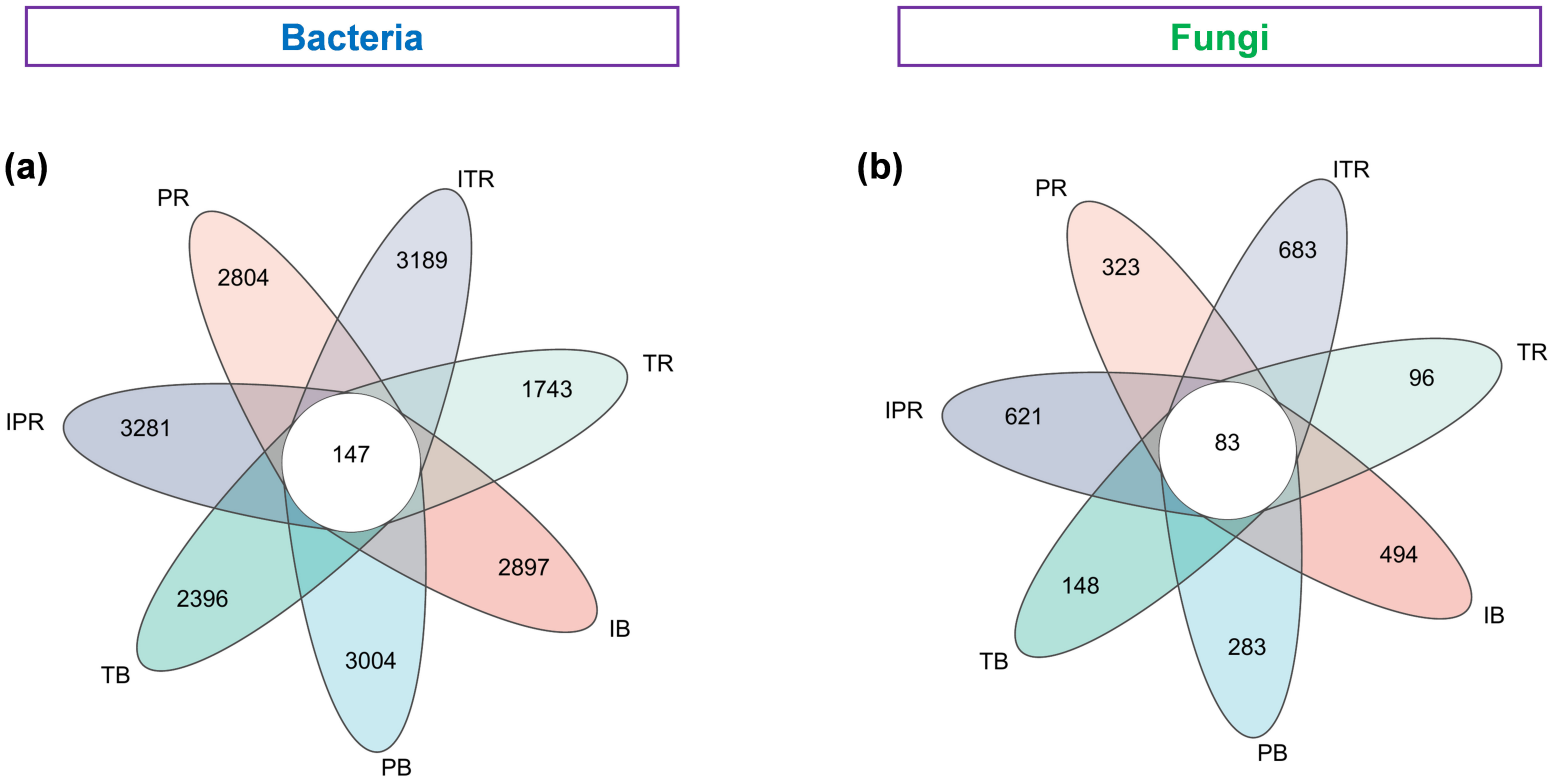
Venn diagram showing the bacterial **(a)** and fungal **(b)** unique and shared ASVs among the seven treated groups based on Bray–Curtis distances at the ASV level. IB: the bulk soil of the *T. grandis* inter-cropping with *P. sibiricum*, PB: the bulk soil for mono-cropping of *P. sibiricum*, TB: the bulk soil for mono-cropping of *T. grandis*, IPR: the *P. grandis* rhizosphere soil of the *T. grandis* inter-cropping with *P. sibiricum*, PR: the rhizosphere soil for mono-cropping of *P. sibiricum*, ITR: the *T. grandis* rhizosphere soil of the *T. grandis* inter-cropping with *P. sibiricum*, and TR: the rhizosphere soil for mono-cropping of *T. grandis*.

In Venn diagrams, 19,458 bacterial ASVs (73.2% of the total ASV) and 1,658 fungal ASVs (65.2% of the total ASV) were used to calculate the distribution of soil bacterial and fungal ASVs in the cropping. The seven samples shared 147 ASVs (0.8%) and 83 ASVs (4.9%) of the bacterial and fungal ASVs. The bacterial and fungal ASVs in the rhizosphere soil of *Torreya-Polygonatum* inter-cropping were higher than those in mono-cropping [bacterial: 3,281 (IPR) > 2,804 (PR), 3,189 (ITR) > 1,743 (TR); fungal: 378 (IPR) > 169 (PR), 401 (ITR) > 88 (TR; Figure 3)].

**Figure 4:**
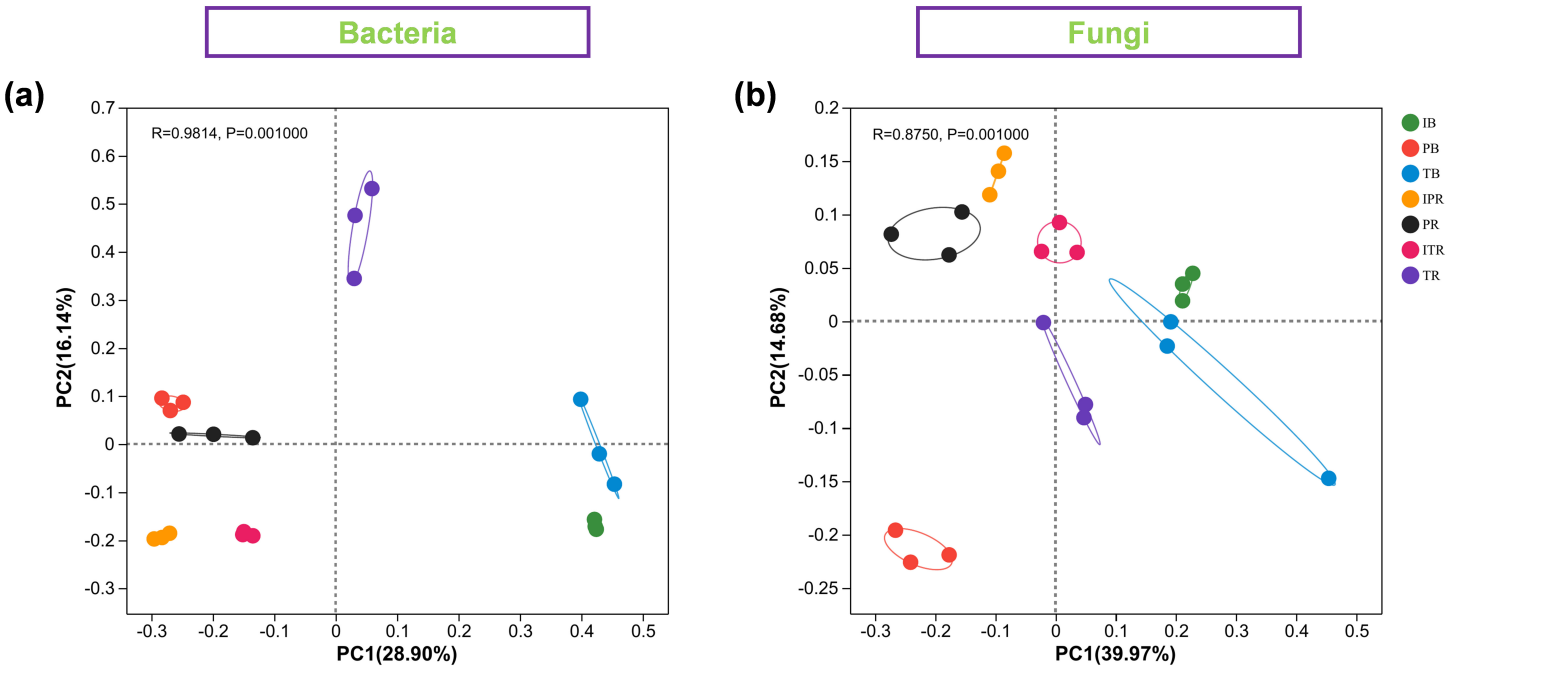
Principal coordinate analysis (PCoA) showing the bacterial **(a)** and fungal **(b)** community structure among the seven sets of samples. IB: the bulk soil of the *T. grandis* inter-cropping with *P. sibiricum*, PB: the bulk soil for mono-cropping of *P. sibiricum*, TB: the bulk soil for mono-cropping of *T. grandis*, IPR: the *P. grandis* rhizosphere soil of the *T. grandis* inter-cropping with *P. sibiricum*, PR: the rhizosphere soil for mono-cropping of *P. sibiricum*, ITR: the *T. grandis* rhizosphere soil of the *T. grandis* inter-cropping with *P. sibiricum*, and TR: the rhizosphere soil for mono-cropping of *T. grandis*.

PCoA based on the Bray–Curtis distance was performed to address the bacterial and fungal community structure of seven samples. The two extracted principal coordinates explained 45.38 and 50.7% of the total variation in bacteria and fungi, respectively (Figure 4). The rest five samples showed separation. The R-values of bacterial and fungal communities were 0.9894 and 0.8786, respectively.

### 3.3 Bacterial and fungal composition diversity under different cropping patterns

**Figure 5:**
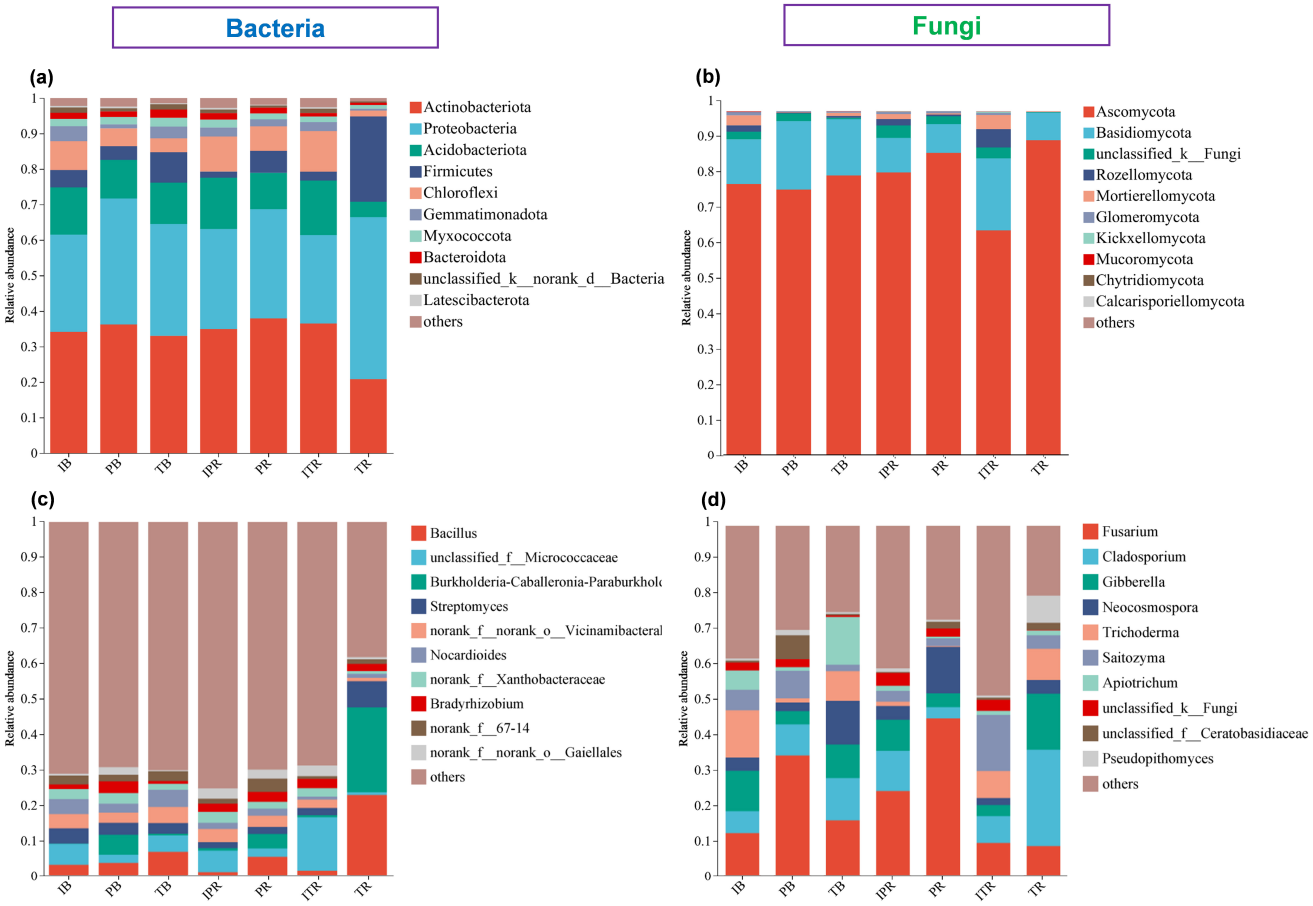
The top 10 most abundant bacterial and fungal groups. **(a, b)** Phyla level at **(a)** bacteria and **(b)** fungi. **(c, d)** Genus level at **(c)** bacteria and **(d)** fungi. IB: the bulk soil of the *T. grandis* inter-cropping with *P. sibiricum*, PB: the bulk soil for mono-cropping of *P. sibiricum*, TB: the bulk soil for mono-cropping of *T. grandis*, IPR: the *P. grandis* rhizosphere soil of the *T. grandis* inter-cropping with *P. sibiricum*, PR: the rhizosphere soil for mono-cropping of *P. sibiricum*, ITR: the *T. grandis* rhizosphere soil of the *T. grandis* inter-cropping with *P. sibiricum*, and TR: the rhizosphere soil for mono-cropping of *T. grandis*.

**Figure 6:**
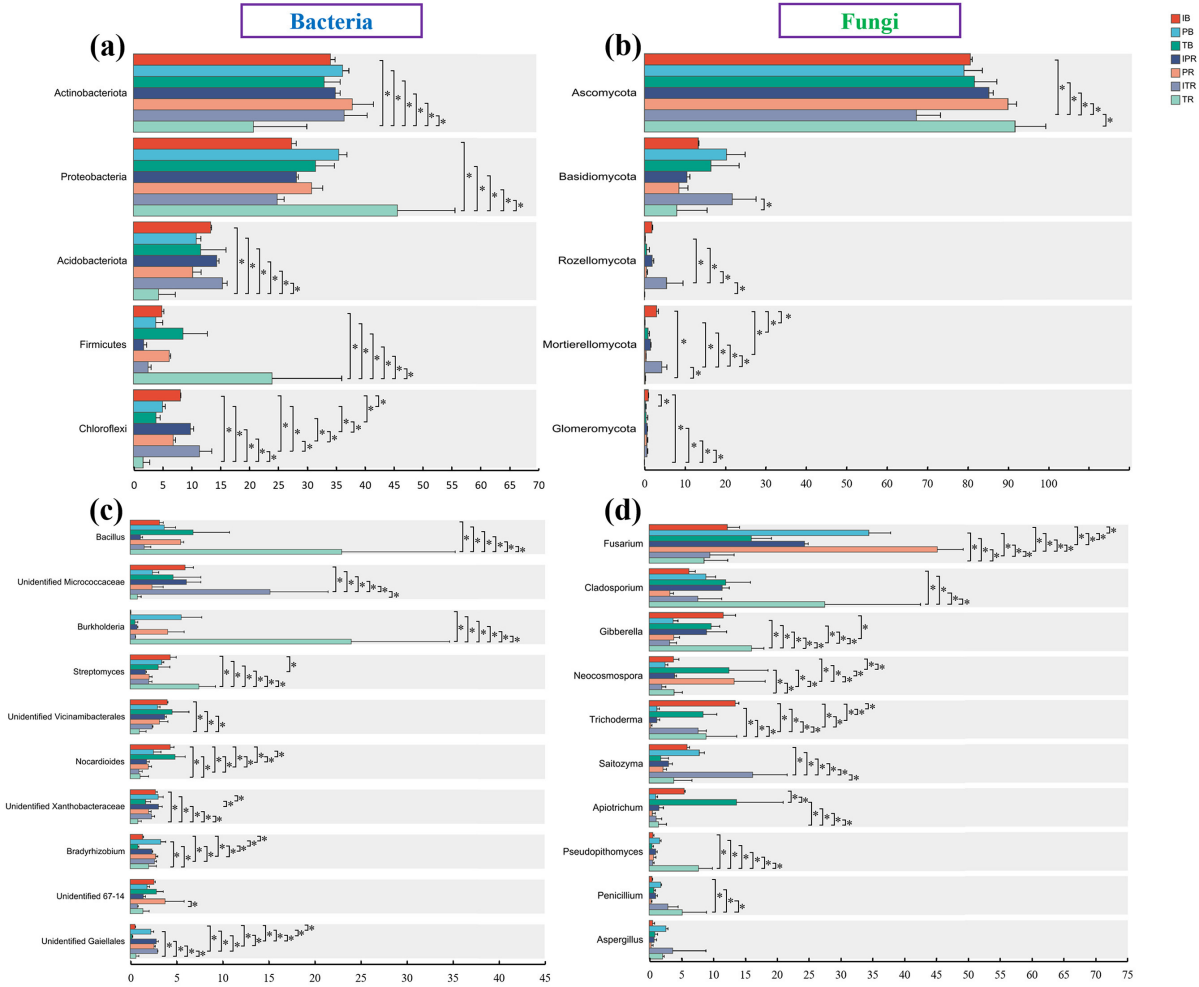
Differences in the abundance of the top bacterial **(a, c)** and fungal **(b, d)**. **(a, b)** Top five phyla level at **(a)** bacteria and **(b)** fungi. **(c, d)** Top 10 genus level at **(c)** bacteria and **(d)** fungi. *Indicate significant differences among the seven sets of samples. IB: the bulk soil of the *T. grandis* inter-cropping with *P. sibiricum*, PB: the bulk soil for mono-cropping of *P. sibiricum*, TB: the bulk soil for mono-cropping of *T. grandis*, IPR: the *P. grandis* rhizosphere soil of the *T. grandis* inter-cropping with *P. sibiricum*, PR: the rhizosphere soil for mono-cropping of *P. sibiricum*, ITR: the *T. grandis* rhizosphere soil of the *T. grandis* inter-cropping with *P. sibiricum*, and TR: the rhizosphere soil for mono-cropping of *T. grandis*.

The dominant bacterial phyla were Actinobacteriota (33.3%), Proteobacteria (32%), Acidobacteriota (11.5%), Firmicutes (7.4%), and Chloroflexi (6.7%) accounted for most (90.9%) of the bacterial sequences (Figure 5a, Supplementary Figure 1). The dominant fungal phyla were Ascomycota (80.6%) and Basidiomycota (13.8%), Rozellomycota (1.4% and Mortierellomycota (1.4%) accounted for the rest (Figure 5b, Supplementary Figure 1). The abundance of the dominant phyla differed across the samples. Compared to TR sample, ITR increased the relative abundance of phyla Actinobacteriota, Acidobacteriota, Chloroflexi, Basidiomycota, Rozellomycota, Mortierellomycota, and Glomeromycota, while Proteobacteria, Firmicutes and Ascomycota significantly decreased (Figures 6a, b). However, except for the higher abundance of Chloroflexi within IPR, there was no significant difference in the abundance of other dominant bacterial and fungal phyla between IPR and PR (Figures 6a, b). For the bulk soil samples, the IB samples showed higher abundance of the Chloroflexi, Mortierellomycota, and Glomeromycota compared to TB sample (IB and PB). Each of the bacterial and fungal phyla in the rhizosphere soil (IPR, PR, ITR, and TR) showed similar abundance. The overall phyla composition remained consistent across the different samples, though the abundance was different (Figures 5a, b, 6).

The most abundant bacterial genus was *Bacillus* (belong to Firmicutes, 6.08% of the sequences), followed by Unidentified Micrococcaceae (Actinobacteriota, 5.24% of the sequences) and *Burkholderia* (Proteobacteria, 4.63% of the sequences), *Streptomyces* (Actinobacteriota, 3.32% of the sequences), and unidentified Vicinamibacterales (Actinobacteriota, 3.17% of the sequences; Figure 5c, Supplementary Figure 2). The top five fungal genera were *Fusarium* (Ascomycota, 23.18% of the sequences), *Cladosporium* (Ascomycota, 10.84% of the sequences), *Gibberella* (Ascomycota, 8.22% of the sequences), *Neocosmospora* (Ascomycota, 6.37% of the sequences), and *Trichoderma* (Ascomycota, 5.84% of the sequences; Figure 5d, Supplementary Figure 2).

Among the top 10 genera of bacteria and fungi with high relative abundance, compared to the TR, ITR increased the relative abundance of unidentified Micrococcaceae, unidentified Xanthobacteraceae, unidentified Gaiellales and *Saitozyma*, while the relative abundance of *Bacillus, Burkholderia, Streptomyces, Cladosporium, Gibberella Apiotrichum*, and *Pseudopithomyces* decreased significantly (Figures 6c, d, Supplementary Tables 3, 4). For two rhizosphere soil of *P. sibiricum* (IPR and PR), *Fusarium and Neocosmospora* had higher abundance in the PR sample (Figures 6c, d, Supplementary Tables 3, 4). For the bulk and rhizosphere soil samples, *Streptomyces, Nocrdioides*, and *Trichoderma* had lower abundance in the rhizosphere soil after inter-croping, while unidentified Gaiellales was higher (IB and IPR, ITR; Figures 6c, d, Supplementary Tables 3, 4). Compare to the TB samples, the TR samples increased the relative abundance of *Bacillus, Burkholderia, Streptomyces, Bradyrhizobium, Gibberella, Neocosmospora*, and *Penicillium*, while the unidentified vicinamibacterales, *Nocardioides, Apiotrichum*, and *Pseudopithomyces* were decreased (Figures 6c, d, Supplementary Tables 3, 4).

### 3.4 Bacterial community composition of predicted function under different cropping patterns

The FAPROTAX classifier facilitates the annotation and prediction of microorganism functions through the construction of a comprehensive database that correlates microbial classification with their respective functionalities. The total number of 2,517 ASVs (9.46% of the total ASV) was assigned to 46 functional bacterial groups based on 16S rDNA sequence classification using FAPROTAX classifier. The dominant functional groups were associated with biogeochemical cyclings, including chemoheterotrophy (33.6%), aerobic chemoheterotrophy (33.3%), and aromatic_compound_degradation (4.7%) involved with C cycle, followed by the groups in the N cycle associated with nitrogen_fixation (3.9%) and nitrate_reduction (1.8%; Supplementary Table 5).

**Figure 7:**
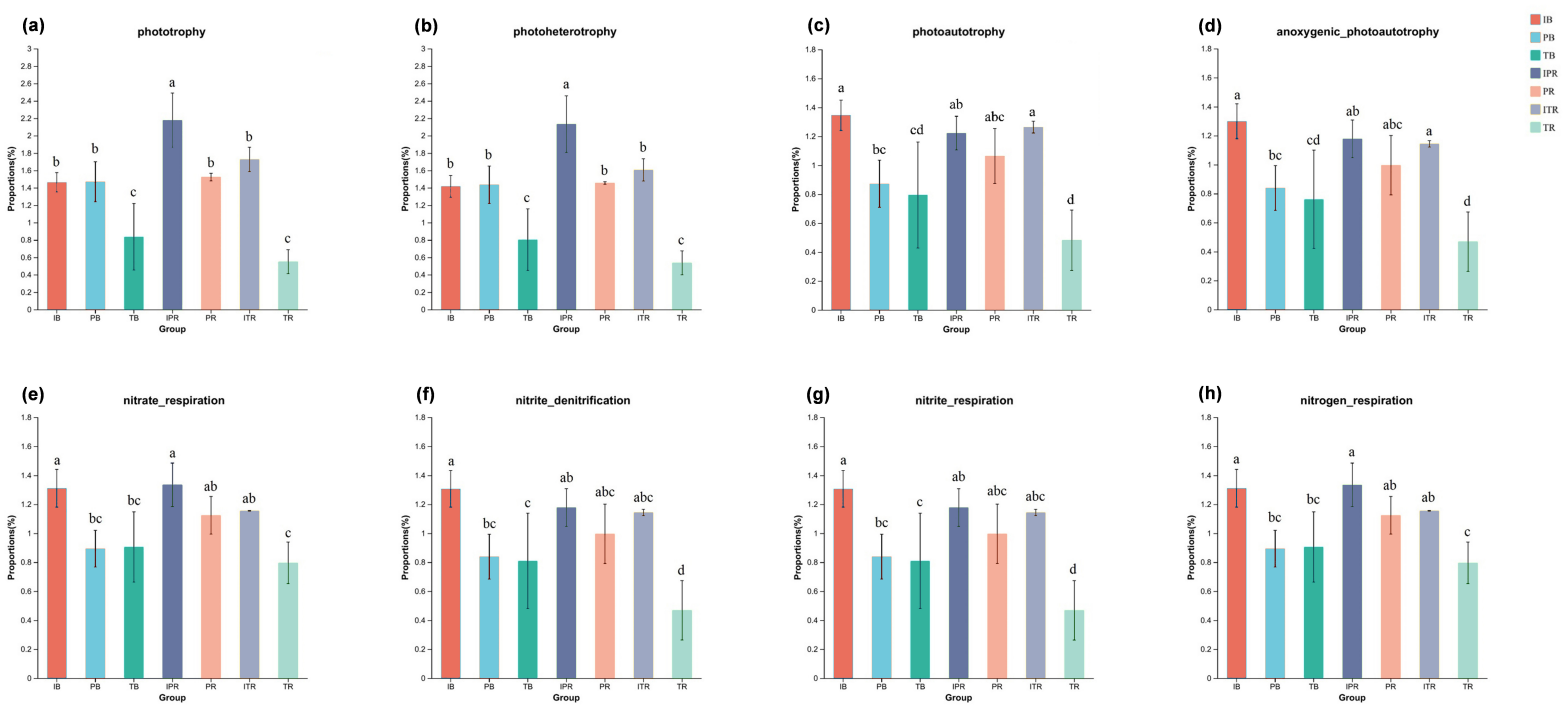
Bacterial ecological functions predicted by FAPROTAX under different cropping practice. Bar chart showing the significantly different functional groups involved in C cycle **(a–d)**, N cycle **(e, h)**. C cycle in phototrophy **(a)**; photoheterotrophy **(b)**; photoautotrophy **(c)**; anoxygenic_photoautotrophy **(d)**; N cycle in nitrate_respiration **(e)**; nitrite_denitrification **(f)**; nitrite_respiration **(g)**; nitrogen_respiration **(h)**. Different lowercase letters indicate significant differences among the seven sets of samples. IB: the bulk soil of the *T. grandis* inter-cropping with *P. sibiricum*, PB: the bulk soil for mono-cropping of *P. sibiricum*, TB: the bulk soil for mono-cropping of *T. grandis*, IPR: the *P. grandis* rhizosphere soil of the *T. grandis* inter-cropping with *P. sibiricum*, PR: the rhizosphere soil for mono-cropping of *P. sibiricum*, ITR: the *T. grandis* rhizosphere soil of the *T. grandis* inter-cropping with *P. sibiricum*, and TR: the rhizosphere soil for mono-cropping of *T. grandis*.

ANOVA analysis disclosed that the functions with significant differences among samples were mainly concentrated in the C cycle (phototrophy, photoheterotrophy, photoautotrophy, and anoxygenic_photoautotrophy, Figures 7a–d) and N cycle (nitrate_respiration, nitrite_denitrification, nitrite_respiration, and nitrogen_respiration, Figures 7e–h) with the same trend. These functions (Figures 7a–h) had the lowest abundance in the TR, of which was significantly lower than that in the ITR. For the bulk and the rhizosphere soil samples, except for the groups of phototrophy, photoheterotrophy, nitrite_denitrification, and nitrite_respiration, there was no significant difference in the other function groups in between.

### 3.5 Fungal community composition of predicted function under different cropping patterns

**Figure 8:**
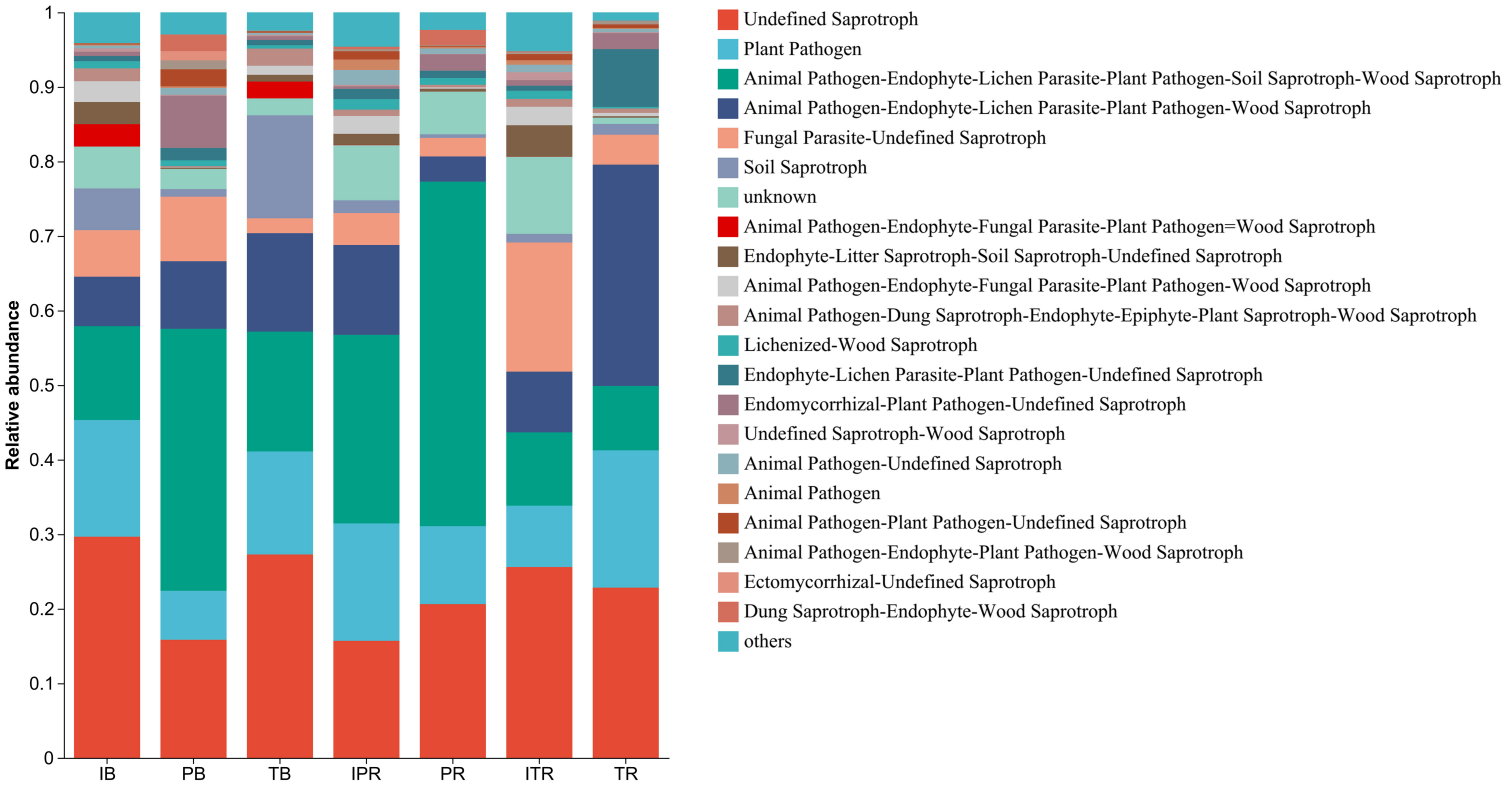
Compositions and relative abundance of assigned fungal functional groups (guild) inferred by FUNGuild. IB: the bulk soil of the *T. grandis* inter-cropping with *P. sibiricum*, PB: the bulk soil for mono-cropping of *P. sibiricum*, TB: the bulk soil for mono-cropping of *T. grandis*, IPR: the *P. grandis* rhizosphere soil of the *T. grandis* inter-cropping with *P. sibiricum*, PR: the rhizosphere soil for mono-cropping of *P. sibiricum*, ITR: the *T. grandis* rhizosphere soil of the *T. grandis* inter-cropping with *P. sibiricum*, and TR: the rhizosphere soil for mono-cropping of *T. grandis*.

FUNGuild was used to predict the function of the fungal community structure of seven samples, incorporating current literature and authoritative website data for categorizing fungi into distinct functional groups based on their nutritional modes. In total, 3,846 ASVs were assigned to different functional groups according to the annotation of ITS sequence classification. According to the absorption and utilization of environmental resources of the fungal community, the fungi were divided into pathotroph, symbiotroph, and saprotroph. Undefined saprotroph was the most enriched functional group, followed by plant and animal pathogens. Fungi are classified into various guilds based on the three major nutritional methods, encompassing animal pathogens, arbuscular mycorrhizal fungi, ectomycorrhizal fungi, lichenicolous fungi, lichenized fungi, plant pathogens, undefined root endophytes, undefined saprotrophs, and wood saprotrophs as the main functional groups. The seven samples had different effects on the abundance of the functional groups. The results further revealed that inter-cropping significantly enhanced the abundance of plant pathogens and Animal Pathogen-Endophyte-Lichen Parasite-Plant Pathogen-Wood Saprotroph in the rhizosphere soil of *P. sibiricum*, conversely, it resulted in a decrease in the abundance of Animal Pathogen-Endophyte-Lichen Parasite-Plant Pathogen-Soil Saprotroph-Wood Saprotroph (Figure 8, Supplementary Table 6). Simultaneously Fungal Parasite-Undefined Saprotroph function groups exhibited an increase in the rhizosphere soil of *T. grandis* after inter-cropping, while plant pathogens and Animal Pathogen-Endophyte-Lichen Parasite-Plant Pathogen-Wood Saprotroph decreased (Figure 8, Supplementary Table 6).

## 4 Discussion

The agricultural productivity and stability rely heavily on the richness and diversity of soil microbial communities (Van Der Heijden et al., 2008). They are often reshaped with different cropping practice. Of which, inter-cropping has been demonstrated to have the potential to enhance beneficial soil microbial communities and effectively mitigate pests and diseases (Zhang et al., 2019; Chai et al., 2021). In this study, we addressed the responses of soil microbial communities to *T. grandis* inter-cropping with *P. sibiricum*, and compared the microbial community disparities between bulk and rhizosphere soil samples.

### 4.1 *Torreya-Polygonatum* inter-cropping improved the soil microbial diversity and communities

Intercropping has the potential to modulate the diversity of soil microbial communities. Previous studies showed that maize inter-cropping with peanut leads to an increase of soil beneficial microbial abundance and diversity (Du et al., 2020; He et al., 2013). Li and Wu (2018) disclosed that wheat inter-cropping with cucumber or mustard with cucumber can enhance the richness of operational microbial taxonomic in soil. In this study, PCoA results revealed that there were distinct variations in bacterial and fungal communities (Figure 4). Compared to mono-cropping, indices of Chao1, Shannon, Shannoneven, and PD using α-diversity analysis were increased both for bulk and rhizosphere soil samples of *T. grandis* inter-cropping with *P. sibiricum* (Figure 2). The study conducted by Deng et al. (2022) found no statistically significant differences in bacterial community richness (Chao1) and diversity (Shannon) between *T. grandis* inter-cropping with *P. sibiricum* and mono-cropping of *T. grandis* (ITR and TR). However, this study revealed a significant variation in the richness and diversity of bacteria and fungi in the rhizosphere soil of *T. grandis* after inter-cropping, while having minimal impact on the composition of bacterial communities in the rhizosphere soil of *P. sibiricum*. Our findings differ from Deng's observations in *T. grandis* plantation, potentially attributed to variations in sampling points (Deng et al., 2022).

### 4.2 *Torreya-Polygonatum* inter-cropping enhanced the roles of key microbial communities

Bacterial phyla of Actinobacteriota, Proteobacteria, Acidobacteriota, Firmicutes, and Chloroflexi were predominant within all samples here (Figure 5). Our results align with previous studies conducted in pure *T. grandis* forest (Feng et al., 2019; Deng et al., 2022) with the exception of Firmicutes. The most predominant phylum Actinobacteriota (33.2%) had significantly higher abundance within *T. grandis* rhizosphere soil after inter-cropping, compared to mono-cropping. The Actinobacteria taxa mainly consist of copiotrophs that strategically thrive on carbon availability and are actively involved with organic matter decomposition and cycling (Liu et al., 2017). Further, *T. grandis* inter-cropping with *P. sibiricum* resulted in relatively elevated presence of Acidobacteriota and Chloroflexi. Taxa residing these two phyla are known to plays crucial roles in polysaccharide degradation as well as the decomposition of non-degradable organic matter (Moghimian et al., 2017; Li et al., 2020). However, the relative abundance of Proteobacteria (represented by *Burkholderia*) and Firmicutes (represented by *Bacillus*) applying inter-cropping was decreased (Figure 6), which is in agreement of previous studies (Gao et al., 2019; Deng et al., 2022).

Inter-cropping known for effectively modulating the soil microbial community can be applied for disease management through reducing pathogen abundance (Zhang et al., 2023). *Cladosporium* speices are crucial plant pathogens responsible for stem rot and leaf spot diseases (Mohamed and Ibrahim, 2021), and in this study, the abundance of *Cladosporium* in the rhizosphere soil of *T. grandis* was significantly decreased following *T. grandis* inter-cropping with *P. sibiricum* (Figure 6d). *Fusarium* is a significant causative agent of root rot in *T. grandis* and *P. sibiricum* (Zhang et al., 2016; Li and Guo, 2019). The similar trend was found on *Fusarium* pathogen within the *P. sibiricum* (Figure 6d). The abundance of the opportunistic pathogen *Neocosmospora* known to induce canker disease, stem and root rot, as well as cane wilt (Zeng and Zhuang, 2023) was also substantially decreased as the result of inter-cropping (Figure 6d). Therefore, it is hypothesized that *T. grandis* inter-cropping with *P. sibiricum* may reduce the abundance of certain pathogens.

Inter-cropping, on the other side, can enhance plant resistance to pathogens and promote plant growth by recruiting beneficial microbial communities (Mendes et al., 2011; Latz et al., 2012; Dhar Purkayastha et al., 2018; Humphrey et al., 2020; Shalev et al., 2022). Xanthobacteraceae comprises a cluster of bacteria closely associated with the carbon cycle, and some species within this group exhibit a facultatively chemolithoautotrophic lifestyle, enabling them to both assimilate and generate carbon dioxide (Kappler et al., 2012). Gemmatimonadaceae species are a group of nitrogen removal bacteria associated with the nitrogen cycle that play a key role in efficient phytic acid mineralization in different ecosystems (Jia et al., 2023). In this study, *T. grandis* inter-cropping with *P. sibiricum* significantly increased the abundance of Xanthobacteraceae and Gemmatimonadaceae in the rhizosphere soil of *T. grandis* (Figure 6c). Based on the analysis and prediction results from FAPROTAX, it can be inferred that intercropping has the potential to enhance the soil microbial communities' capacity for carbon and nitrogen cycling, thereby facilitating nutrient circulation within plants. The probiotic Saitozyma present in soil and plant rhizosphere exhibits the potential to enhance plant growth and disease management (Das et al., 2023). In this study, the rhizosphere soil of *T. grandis* was significantly improved after inter-cropping practice, suggesting its ability to recruit beneficial microorganisms for growth and disease control.

### 4.3 The influence of *Torreya-Polygonatum* inter-cropping on potential microbial function alteration

Changes in microbial community structure can result in microbial functional alterations (Preston-Mafham et al., 2002). FAPROTAX was employed to predict the functional profiles of soil bacteria under different samples. The results revealed that chemoheterotrophy (33.6%) and aerobic chemoheterotrophy (33.3%) were the predominant enriched functions, consistent with previous findings (Rocha et al., 2020; Wang et al., 2023). Previous studies disclosed that inter-cropping significantly enhanced soil microbial function associated with nutrient cycling and exhibited a consistent increase in the relative abundance of nitrogen-cycling and carbon-cycling bacteria (Zhong et al., 2022; Marcos-Pérez et al., 2023). In our study, we found that genes involved in carbon cycling (phototrophy, photoheterotrophy, photoautotrophy, and anoxygenic_photoautotrophy) and nitrogen cycling (nitrate_respiration, nitrite_denitrification, nitrite_respiration, and nitrogen_respiration) exhibited significantly higher abundance in the ITR sample compared to TR. These results suggest that *T. grandis* inter-cropping with *P. sibiricum* may enhance nutrient cycling in soil through its influence on bacterial functionality and alteration of soil bacterial community composition. This could be attributed to feedback cycles facilitated by C-cycle bacteria (Xanthobacteraceae) and N-cycle bacteria (Gemmatimonadaceae), which showed a significant increase under the inter-cropping samples.

FUNGuild was used to achieve fungal functional determination (Song et al., 2017; Nguyen et al., 2016). Undefined saprotrophs as the most abundant fungal functional group, may be attributed to the high prevalence of ascomycetes in each sample group, given that a significant portion of fungi within this phylum are associated with organic decomposition processes (Nguyen et al., 2016). Inter treatment comparisons reveal a lower prevalence of plant pathogen guild in the intercropped sample ITR compared to the TR, which aligns with the reduced abundance of pathogenic fungi such as *Cladosporium, Gibberella*, and *Neocosmospora*in in ITR, further contributes to mitigating soil-borne plant pathogens, thereby enhancing crop yield and disease resistance.

## 5 Conclusion

This study focused the impact of *T. grandis* inter-cropping with *P. sibiricum* and mono-cropping on soil microbial diversity and function. The results demonstrate that *T. grandis* inter-cropping with *P. sibiricum* significantly increases the abundance and diversity of soil fungal and bacterial communities comparing to mono-cropping. Moreover, this agricultural practice also enhanced the microbial functionality pertaining to nitrogen and carbon cycles, thereby potentially facilitating nutrient uptake by plants while concurrently reducing the prevalence of specific pathogens. The findings arisen from the study are helpful for agroforestry sustainable development and effective management.

## Data Availability

The datasets presented in this study can be found in online repositories. The names of the repository/repositories and accession number(s) can be found at: https://www.ncbi.nlm.nih.gov/, PRJNA1150097.
