# Supplementary material for: The effect of Torreya grandis inter-cropping with Polygonatum sibiricum on soil microbial community
Source: Front Microbiol. 2024 Dec 4;15:1487619. doi: 10.3389/fmicb.2024.1487619 (PMC11652488; doi:10.3389/fmicb.2024.1487619)
Supplement: Supplementary file 1 [file Table_1.docx]

**Table. S1.** The site information of the seven sets of samples

| Site | Chun’an County, Hangzhou, China |
| --- | --- |
| GPS | 29°11′46″N, 118°42′10″E |
| Average annual sunshine | 1850.3 hours |
| Average annual precipitation | 1515.1 mm |
| Average temperature | 17.2°C |
