# Supplementary material for: The effect of Torreya grandis inter-cropping with Polygonatum sibiricum on soil microbial community
Source: Front Microbiol. 2024 Dec 4;15:1487619. doi: 10.3389/fmicb.2024.1487619 (PMC11652488; doi:10.3389/fmicb.2024.1487619)
Supplement: Supplementary file 2 [file Table_2.docx]

|  | Sample | Chao1 | Shannon | Shannoneven | Pd | Coverage |
| --- | --- | --- | --- | --- | --- | --- |
| Bacteria | IB | 474.52±10.05ab | 4.75±0.01a | 0.77±0.004a | 142.50±2.77ab | 99.97% |
|  | PB | 435.46±8.45b | 4.79±0.04a | 0.79±0.007a | 126.22±1.76de | 99.97% |
|  | TB | 437.88±10.89b | 4.57±0.14a | 0.75±0.02a | 128.51±2.92cd | 99.97% |
|  | IPR | 513.86±3.53a | 4.89±0.02a | 0.78±0.003a | 147.85±0.78a | 99.96% |
|  | PR | 464.95±9.66ab | 4.75±0.05a | 0.77±0.007a | 131.34±2.92bcd | 99.97% |
|  | ITR | 466.15±12.61ab | 4.47±0.12a | 0.72±0.017a | 139.55±2.35abc | 99.98% |
|  | TR | 381.70±36.70c | 3.30±0.45b | 0.55±0.066b | 114.25±9.07e | 99.99% |
| Fungi | IB | 210.67±6.01bc | 3.32±0.04a | 0.62±0.006ab | 65.07±3.24bc | 100% |
|  | PB | 174.00±7.94cde | 2.93±0.07b | 0.57±0.008bc | 53.91±2.8cd | 100% |
|  | TB | 151.67±8.65de | 2.82±0.17bc | 0.56±0.029bc | 47.5±3.33de | 100% |
|  | IPR | 269.00±3.06a | 3.47±0.06a | 0.62±0.011ab | 81.86±1.02a | 100% |
|  | PR | 184.67±27.85cde | 2.49±0.05c | 0.48±0.014d | 60.18±7.84c | 100% |
|  | ITR | 234.67±8.88ab | 3.6±0.04a | 0.66±0.01ab | 74.33±1.09ab | 100% |
|  | TR | 137.00±6.43e | 2.54±0.21c | 0.52±0.037cd | 41.48±3.05e | 100% |

**Table. S2.** Alpha diversity indices of the seven treated groups
