# Supplementary figures and images for: The effect of Torreya grandis inter-cropping with Polygonatum sibiricum on soil microbial community
Source: Front Microbiol. 2024 Dec 4;15:1487619. doi: 10.3389/fmicb.2024.1487619 (PMC11652488; doi:10.3389/fmicb.2024.1487619)

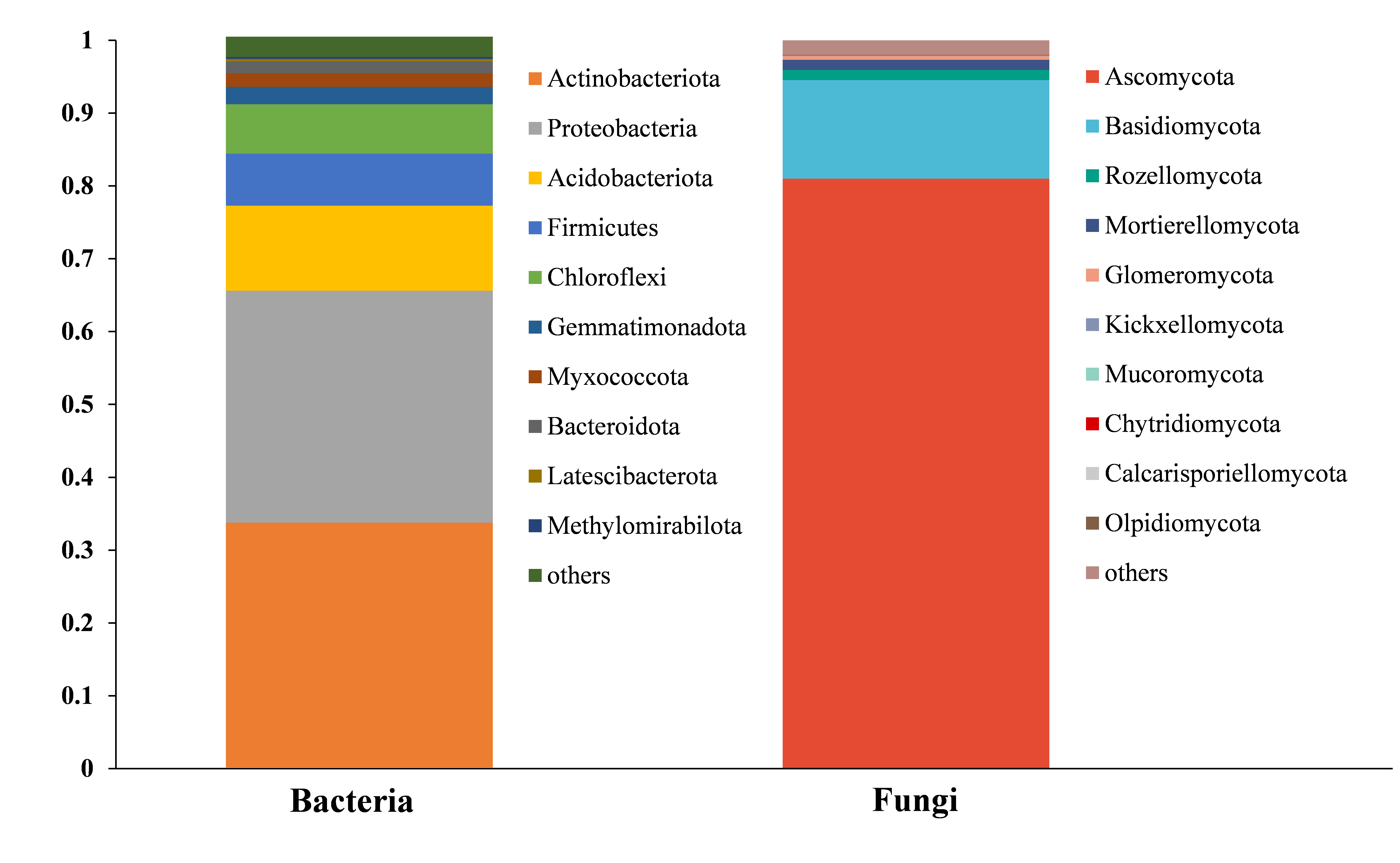

Supplement: Supplementary file 7 [file Image_1.jpeg]

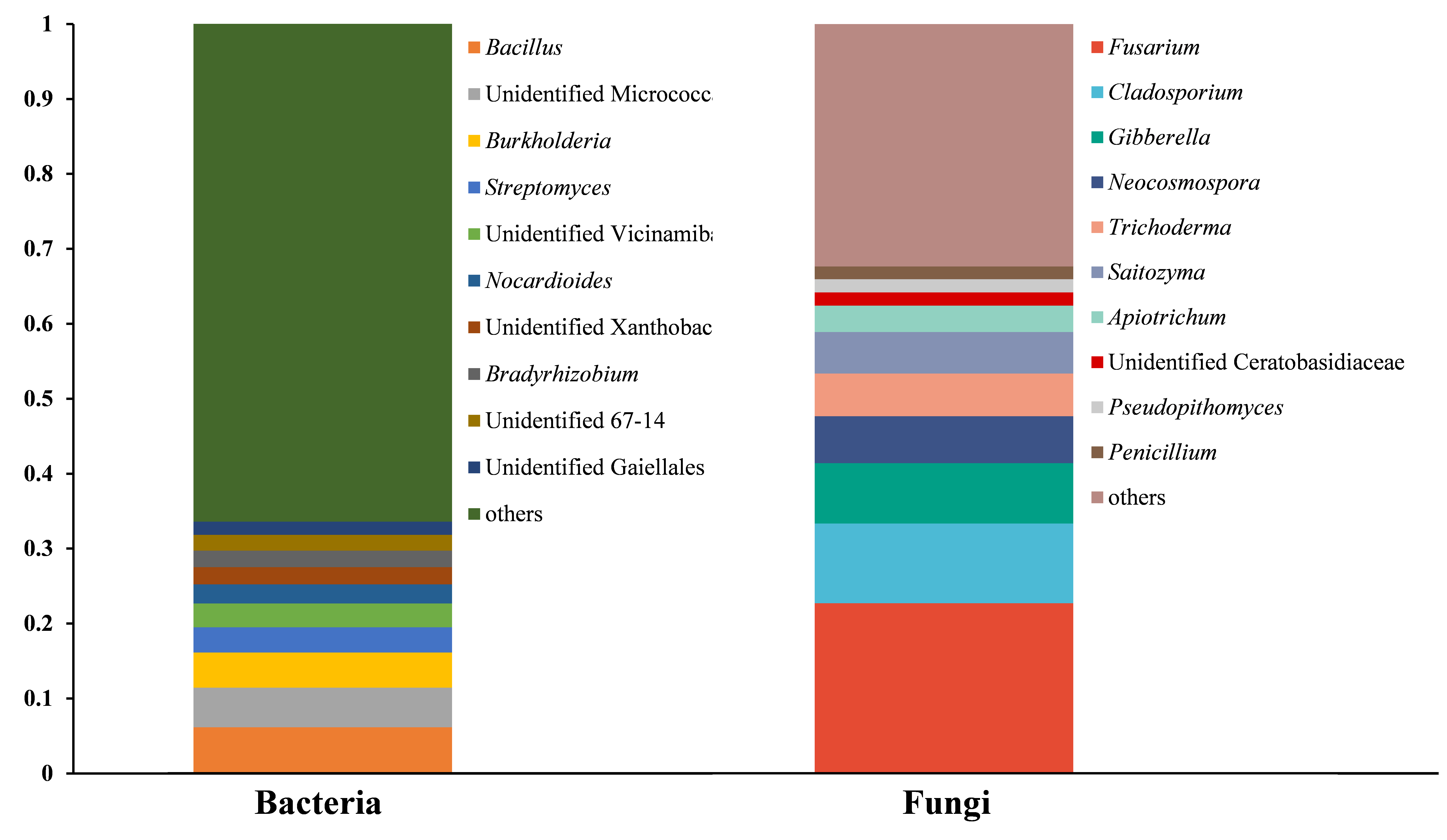

Supplement: Supplementary file 8 [file Image_2.jpeg]

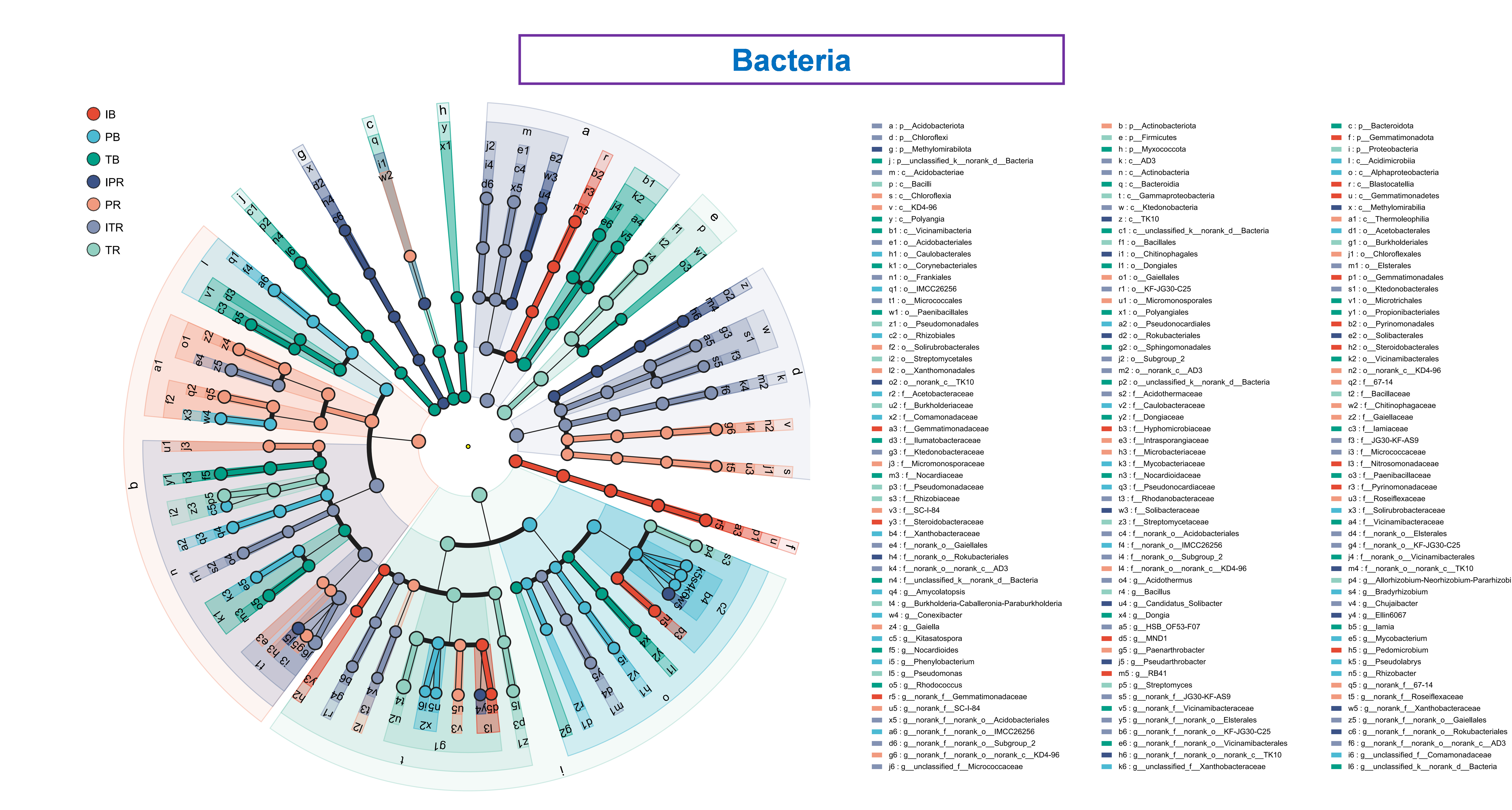

Supplement: Supplementary file 9 [file Image_3.jpeg]

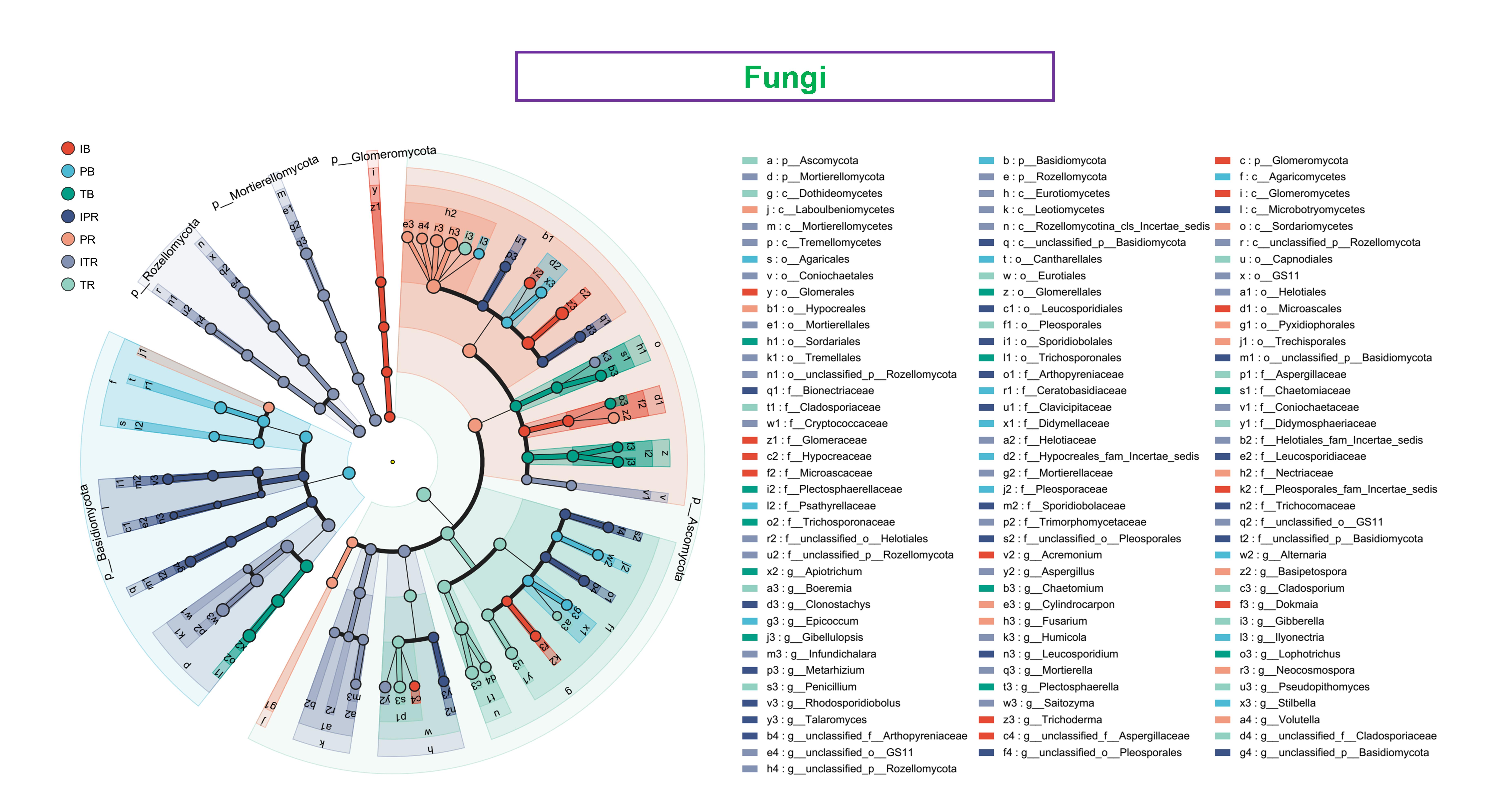

Supplement: Supplementary file 10 [file Image_4.jpeg]
